# PI3Kα inhibitor impairs AKT phosphorylation and synergizes with novel angiogenesis inhibitor AL3810 in human hepatocellular carcinoma

**DOI:** 10.1038/s41392-021-00522-6

**Published:** 2021-03-31

**Authors:** Qin Xie, Shuaishuai Chi, Yanfen Fang, Yiming Sun, Linghua Meng, Jian Ding, Yi Chen

**Affiliations:** 1 College of Pharmaceutical Sciences, Zhejiang University, Hangzhou, People’s Republic of China; 2 Division of Anti-Tumor Pharmacology, Shanghai Institute of Materia Medica, Chinese Academy of Sciences, Shanghai, People’s Republic of China; 3 University of Chinese Academy of Sciences, Beijing, People’s Republic of China; 4 State Key Laboratory of Drug Research, Shanghai Institute of Materia Medica, Chinese Academy of Sciences, Shanghai, People’s Republic of China; 5 Shanghai HaiHe Pharmaceutical Co. Ltd, Shanghai, People’s Republic of China

**Keywords:** Cancer microenvironment, Drug development

**Dear Editor**,

Hepatocellular carcinoma (HCC) is the second leading cause of cancer-related death worldwide with a 5-year survival rate ~50–80% after curative treatment, like surgery, targeted therapy plus TACE or RFA or local treatments, immunotherapy, and so on.^1^ The approval of several angiogenesis inhibitors, such as sorafenib, regorafenib, lenvatinib, and cabozantinib, have shown therapeutic potential for HCC treatment. However, the improvement of overall survival or progression-free survival is still limited based on angiogenesis inhibitor monotherapy at present.^2^ Therefore, novel compounds and rational combinations are urgently required. AL3810 was discovered as a novel and orally bioavailable small-molecule angiogenesis inhibitor by our group targeting VEGFR, PDGFR, and FGFR, having potent antiangiogenic and antitumor efficacy in multiple tumors.^3,4^ Here, we aim to comprehensively exploit the potential combination opportunity for AL3810 and PI3Kα inhibitor (PI3Kαi) CYH33 against HCC, and clarify the possible mechanism to support the combination in clinical application. CYH33 is a novel, highly selective PI3Kα inhibitor, also discovered by our group and currently in clinical trials (NCT03544905).^5^

First, HCC cell lines Bel-7402 and SMMC-7721 were exposed to AL3810 with or without the specific PI3Kα inhibitors CYH33 or BYL719, the first approved specific PI3Kα inhibitor for breast cancer. As expected, the remarkable synergistic anti-proliferation efficacy was displayed in “heat” graphs (Supplementary Fig. S1a). The dose-response curves also suggested robustly synergistic anti-proliferation efficacy in JHH7, Huh7, Bel-7402, and SMMC-7721 treated with AL3810 and PI3Kαi alone or combination (Supplementary Fig. S1b). Then, 22 HCC cell lines and a human HCC patient-derived cell line (PDC) CLC2 were expanded to evaluate the combined effect by calculating combination index (CI) values. Almost all CI values were <0.8, indicating the potent synergistic efficacy of AL3810 and PI3Kαi (Fig. 1a, b). Here, sorafenib also exerted the synergistic effect with PI3Kαi against HCCs proliferation. The IC_50_ values of all the co-treatment groups apparently were less than AL3810 or sorafenib alone in tested 22 HCCs (Supplementary Fig. S1c, d). AL3810 and PI3Kαi also synergistically inhibited the colony formation in multiple HCCs (Supplementary Fig. S1e). To further assess the synergistic antitumor efficacy in vivo, HCC cell line-derived (Bel-7402, Huh7, JHH7, and SMMC-7721) xenograft (CDX) models, and HCC patient-derived xenograft (PDX) models were utilized. The tumor growth was significantly retarded in the combination group in all tested CDX and PDX models (Fig. 1c and Supplementary Fig. S1f). The mice’s body weight had almost no changes (Supplementary Fig. S1g).

**Fig. 1:**
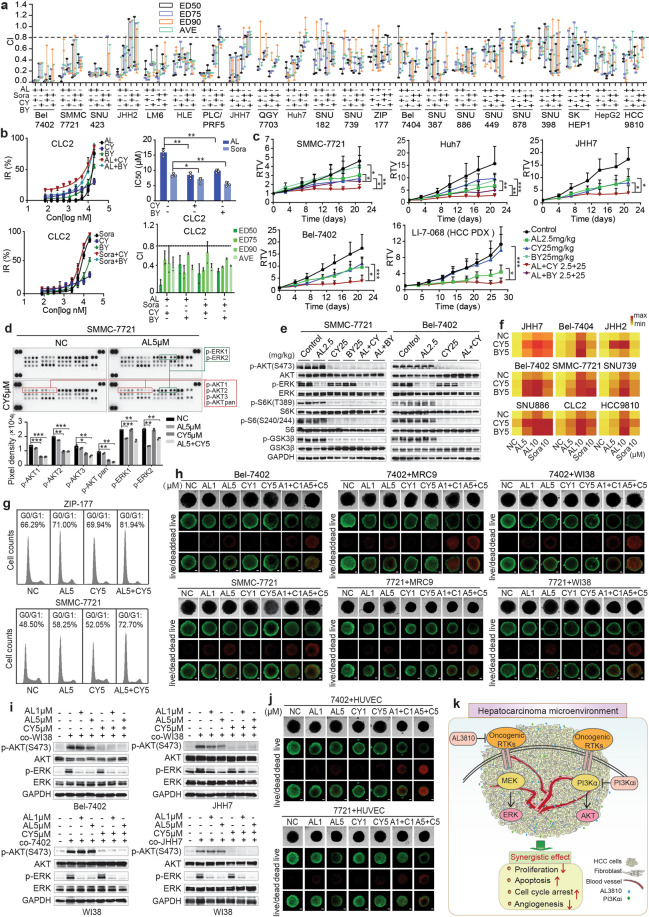
**a** Twenty-two hepatocellular carcinoma (HCC) cell lines were treated with AL3810, sorafenib, CYH33, and BYL719 alone or in combination with a series of concentrations starting at 20 μM in twofold dilution for 3 days. The combination index (CI) of multiple HCC cells after exposure to AL3810, sorafenib alone or combined with CYH33 or BYL719 were calculated by CalcuSyn software. **b** Human HCC PDC CLC2 was incubated with AL3810, sorafenib alone, or combined with CYH33/BYL719 at the starting concentration of 20 μM with twofold dilution for 3 days. The CI values were calculated. **c** The synergistic efficacy of AL3810 combined with CYH33 or BYL719 in vivo was evaluated in HCC cell lines-derived (SMMC-7721, JHH7, Huh7, and Bel-7402) xenograft (CDX) models and patient-derived HCC (LI-7-068) xenograft (PDX) model respectively. The data are displayed as mean + SD. Relative tumor volume (RTV) was calculated as: (½ × length × width^2^ of day *n*) / (½ × length × width^2^ of day 0). **d** The proteome profiler human phospho-MAPK array was used to detect the expression of the indicated proteins phosphorylation in the MAPK pathways in SMMC-7721 cells treated with 5 μM AL3810 and 5 μM CYH33 alone or both for 2 h (up panel), and quantitative pixel densities were shown in the down panel. **e** Western blot analyses the expression levels of relative proteins in SMMC-7721 and Bel-7402 xenograft tumor tissues treated with AL3810, PI3Kαi, or both. **f** A set of HCC cell lines were treated with 5 μM or 10 μM AL3810/sorafenib and 5 μM CYH33/BYL719 alone or in combination for 72 h. The apoptosis rate was assessed by Annexin V/PI double-staining and the quantitative results were presented in a heatmap with min and max apoptosis rate (%) ranging from 1 to 99% correspondingly. **g** The cell cycle of ZIP-177 and SMMC-7721 cells were assessed via flow cytometry after treatment with 5 μM AL3810 and 5 μM CYH33 alone or combined for 48 h. **h** Bel-7402 and SMMC-7721 cells were co-cultured with fibroblast cells MRC9 or WI38 to form the 3D tumor spheroids in the ratio of 4:1, and then administrated with AL3810 combined with CYH33 for 72 hours. The live/dead function was measured via a live/dead viability/cytotoxicity kit. Scale bar indicates 100 μm. **i** Bel-7402 and JHH7 cells were starved for 24 h and then co-cultured with fibroblast cell lines WI38 for another 24 h. AKT and ERK pathways were evaluated by western blot, as well as fibroblast cell WI38 co-cultured with Bel-7402 and JHH7 cells. **j** Bel-7402 and SMMC-7721 cells were co-cultured with HUVECs treated with AL3810 and CYH33 for 72 h. The live/dead viabilities were measured via a live/dead viability/cytotoxicity kit. Scale bar indicates 100 μm. **k** Schematic diagram depicting the combined regulation by AL3810 and PI3Kα inhibitors in the HCC microenvironment. Compound AL3810 collaborates with PI3Kαi synergistically exerting proliferation inhibition, inducing apoptosis, cell cycle arrest, and angiogenesis suppression depending on dual-signaling cascade blockade of MAPK-ERK and PI3K-AKT in the HCC microenvironment. PI3Kαi PI3Kα inhibitors

We then examined the synergistic effect of AL3810 and PI3Kαi on tyrosine kinase signaling pathways. The relative phosphorylation of 26 different human mitogen-activated protein kinases in SMMC-7721 cells treated with AL3810, CYH33, or both was determined by the human phospho-MAPK (p-MAPK) array kit. The quantitative pixel densities showed AL3810 alone only selectively inhibited p-ERK and were resistant to the AKT pathway, while CYH33 alone specifically suppressed p-AKT. The phosphorylation of ERK and AKT was blocked simultaneously after combination treatment (Fig. 1d). Western blot results identified AL3810 exclusively inhibited p-ERK without affecting p-AKT in 11 HCCs. Consistent with AL3810, sorafenib also only selectively decreased p-ERK even at higher doses up to 15 μM. CYH33 and BYL719 selectively suppressed p-AKT (Supplementary Fig. S2a, b). However, the dual suppression of AKT and ERK pathways could be observed at 0.5 h after treatment with the combination therapy, and last for 24 h in tested HCC cells treated with 5 μM AL3810 and 5 μM CYH33 (Supplementary Fig. S2c, d). The dual suppression was also observed after treatment with the combination in SMMC-7721 and Bel-7402 xenografts (Fig. 1e). In addition, as expected, CI values of AL3810 or sorafenib combined with AKT inhibitors GDC0068 and MK2206 were universally less than 0.8 (Supplementary Fig. S3a). Compared to the negative control, the depletion of ERK (1, 2, or 1 + 2) or AKT (1, 2, 3, or 1 + 2 + 3), respectively, all partially reversed the combination inhibition rate of AL3810 and CYH33 in HCCs (Supplementary Fig. S3b, c). Moreover, when HCCs were given MK2206 to repress the AKT pathway, the combination inhibition rate of AL3810 and CYH33 were partially counteracted (Supplementary Fig. S3d). All these demonstrate AL3810 combined with PI3Kαi synergistically suppress the growth of HCCs depending on dual-blocking MAPK-ERK and PI3K-AKT pathways.

AL3810 or sorafenib synergized with PI3Kαi displayed a dramatic apoptosis accumulation compared to mono-compound in a panel of HCCs as shown in heat graphs (Fig. 1f). Western blot further revealed the combination significantly increased cleaved PARP and decreased XIAP in HCCs (Supplementary Fig. S4a). TUNEL staining manifested AL3810 or PI3Kαi alone induced moderate apoptosis, while their combination significantly increased apoptosis population in HCC xenografts (Supplementary Fig. S4b). In addition, G1-phase cells in the dual-treated group were dramatically elevated (Fig. 1g and Supplementary Fig. S4c), accompanied by more p27 upregulation and CyclinD1 downregulation, two typical G1 regulators (Supplementary Fig. S4d).

Three-dimensional (3D) tumor spheroid cell cultures deliver more accuracy to reflect the complexity and heterogeneity mimicking tumor microenvironment in vivo and are more appropriate for the evaluation of angiogenesis inhibitors. We further used 3D culture containing both cancer cells and tumor microenvironment (TME) cells to evaluate the combined effect. Initially, we generated the 3D spheroids of HCCs co-cultured with fibroblasts WI38 or MRC9, which was verified with mCherry and EGFP (Supplementary Fig. S5a–c). 3D tumor spheroid viability was also synergistically suppressed following AL3810 or sorafenib combined with PI3Kαi (Supplementary Fig. S5d). Further, combined therapy resulted in a dramatic increase in dead spheres by live/dead viability/cytotoxicity kit assay (Fig. 1h). Then, the ERK and AKT activities were examined in HCC cells co-cultured with fibroblasts WI38. AL3810 also inhibited WI38-induced p-ERK1/2 in HCCs. The ERK and AKT pathways were still dual-blockaded after treated with the combination. Similar results were got in WI38 co-cultured with HCCs (Fig. 1i). The immunohistochemical quantified results showed that α-SMA, the fibroblast activity indicator, in HCC xenografts was sharply reduced in the combination groups (Supplementary Fig. S5e).

Then, we attempt to explore whether the two kinds of antitumor drugs have the synergetic capacity of anti-angiogenesis efficacy via several steps. The combination resulted in a synergistic inhibition against HUVEC proliferation with CI values <0.8 (Supplementary Fig. S6a). Further, AL3810 plus CYH33 significantly enhanced anti-angiogenetic effects identified by inhibiting tube formation, reducing HUVEC migration, and suppressing rat aortic ring sprouting (Supplementary Fig. S6b–d). Meanwhile, 3D tumor spheroids assay (HCC cells with HUVECs, Supplementary Fig. S6e) displayed that AL3810 synergized with CYH33 resulting in enhanced spheroid dead levels compared with the mono-compound group (Fig. 1j). The angiogenesis maker CD31 accumulation was dramatically reduced in the combination therapy group in vivo (Supplementary Fig. S6f). Notably, the set of experiments verify AL3810 not only has its own anti-angiogenesis activity but also exert synergetic angiogenesis suppression combined with PI3Kαi, closely correlated with its antitumor effect.

Totally, our observations explicitly demonstrate that a combination of novel PI3Kα inhibitors with AL3810 displayed synergistic activity against HCC in vitro and in vivo by dual-inhibition of AKT and ERK phosphorylation in HCCs and TME (Fig. 1k). This is the first attempt to prove AL3810 combined with PI3Kα inhibitor is a reasonable treatment strategy, and provide a mechanistic rational to test AL3810 and other angiogenesis inhibitors in combination with PI3Kαi for treating HCC in future clinical trials. These results also provide a new possibility for the clinical application of AL3810 and CYH33.

## Supplementary information

Supplementary materials

## References

[1] Llovet JM (2016). Hepatocellular carcinoma. Nat. Rev. Dis. Prim..

[2] Finn RS (2018). Therapies for advanced stage hepatocellular carcinoma with macrovascular invasion or metastatic disease: a systematic review and meta-analysis. Hepatology.

[3] Zhou YF (2012). AL3810, a multi-tyrosine kinase inhibitor, exhibits potent anti-angiogenic and anti-tumour activity via targeting VEGFR, FGFR and PDGFR. J. Cell Mol. Med..

[4] Xie Q (2017). Evaluation of in vitro and in vivo activity of a multityrosine kinase inhibitor, AL3810, against human thyroid cancer. Acta Pharm. Sin..

[5] Shi JJ (2019). PI3Kalpha inhibitors sensitize esophageal squamous cell carcinoma to radiation by abrogating survival signals in tumor cells and tumor microenvironment. Cancer Lett..

