# Supplementary material for: PI3Kα inhibitor impairs AKT phosphorylation and synergizes with novel angiogenesis inhibitor AL3810 in human hepatocellular carcinoma
Source: Signal Transduct Target Ther. 2021 Mar 31;6:130. doi: 10.1038/s41392-021-00522-6 (PMC8010115; doi:10.1038/s41392-021-00522-6)
Supplement: Supplementary file 1 — Supplementary materials [file 41392_2021_522_MOESM1_ESM.docx]

**Supplementary materials for**

PI3Kα inhibitor impairs AKT phosphorylation and synergizes with novel angiogenesis inhibitor AL3810 in human hepatocellular carcinoma

Qin Xie^1,2^, Shuaishuai Chi^2,3^, Yanfen Fang^2^, Yiming Sun^2^, Linghua Meng^2^, Jian Ding^2,4,5★^, Yi Chen^2,3★^

Correspondence to: Yi Chen, Email: ychen@simm.ac.cn

Jian Ding, Email: [jding@simm.ac.cn](mailto:jding@simm.ac.cn)

This file includes:

Materials and Methods

Supplementary figures S1 to S6

Supplementary Tables S1 to S4

**MATERIALS AND METHODS**

Compounds

AL3810 and CYH33 were provided by Shanghai HaiHe Pharmaceutical Co., Ltd (Shanghai, China) with purity of 99.1% and 99.9% respectively. BYL719 was purchased from MedChemExpress (Monmouth Junction, NJ, USA). Sorafenib, GDC0068 and MK2206 were purchased from Selleck (Houston, TX, USA). For *in vitro* studies, these compounds were dissolved in DMSO as a stock solution in 10mM or 20mM stored at -80°C. For *in vivo* studies, AL3810 was dissolved in 0.5% carboxymethyl cellulose sodium; CYH33 and BYL719 were dissolved in 0.5% carboxymethyl cellulose sodium with 0.5% tween 80.

Cell cultures

The source and culture condition of 22 hepatocellular carcinoma cell lines, human primary hepatic carcinoma cells, human umbilical vein endothelial cells (HUVEC) and two fibroblast cells WI38 and MRC9 are described in supplementary table S1. All these cells were cultured following the providers’ instructions.

Cell proliferation assay

Hepatocellular carcinoma cells were seeded at an appropriate density of 3000-5000 cells per well in 96-well plates and maintained for 24 hours before exposure to increasing gradient doses of AL3810, sorafenib, CYH33 and BYL719. After being cultured for 3 days, hepatocellular carcinoma cells were fixed with 10% trichloroacetic acid (TCA) overnight, then stained with Sulforhodamine B (Sigma Aldrich, Darmstadt, Hesse, Germany) in 1% acetic acid (v/v) for 20-30 min. Lastly, SRB stained in the cells was dissolved with 150μL of 10mM Tris-HCl and measured at 560nm wavelength by a SpectraMax plus384 (Molecular Devices, Sunnyvale, CA, USA). The dosages corresponding to the half-maximal inhibition (IC_50_) were calculated using a SoftMax pro-based nonlinear 4-parameter regression analysis. IC_50_ values were presented in histograms with mean ± SD. To determine the synergistic effect, combination index (CI) was calculated by CalcuSyn software. When CI value is less than 0.8, indicates a significant synergy effect. When CI value is ranged from 0.8 to 1.2, indicates an additive effect. And when CI value is more than 1.2, indicates an antagonistic effect.

Western blot assay

Cells were cultured with compounds at indicated doses and time respectively. And equal amount of proteins were probed with primary antibodies which were listed in the supplementary table S2 at 4°C overnight, then incubation with corresponding second antibody. Pictures were captured by ImageQuant LAS 4000 (General Electric, Boston, MA, USA).

Phosphorylation of MAPK (phospho-MAPK) pathways array assay

The proteome profiler human phospho-MAPK array kit (R&D Systems, Minneapolis, MN, USA) was used to determine the phosphorylation levels of the major MAPKs families according to the recommended instructions.

Small interfering RNA (siRNA)

HCC cells were seeded in 6-well plates at the density of 2×10^5^ per well for 24 hours. The following day, 5μL RNAi MAX transfection reagent in 250μL opti-MEM was mixed with 5μL corresponding siRNA in 250μL opti-MEM for 15min at room temperature, and then incubated with cells for 48 hours. The sequences used for siRNA are listed in supplementary table S4.

Annexin V/PI apoptosis assay

HCC cells were seeded in 6-well plates at the density of 1.5~2×10^5^ per well and cultured in medium alone or with various concentrations of indicated compounds for 72 hours. An Annexin V-FITC/PI double-staining apoptosis detection kit (Vazyme Biotech, Nanjing, China) was utilized to detect the percentage of cell apoptosis quantitatively. Cells were collected and dyed according to the instruction, and then probed with the FACS Calibur apparatus (BD Biosciences, Franklin lake, NJ, USA). The proportion of cell apoptosis was analyzed with the FlowJo software.

Cell cycle assay

Briefly, HCC cells were collected and stained with RNAse A and PI at room temperature for 30 min, and then detected by the FACS Calibur apparatus. In general, 10000 cells were acquired, and the cell cycle results were analyzed with the BD Cell Quest Pro modify software.

Colony formation assay

HCC cells were cultured at the density of 500-1000 cells per well in 6-well plates with indicated compounds for 10-15 days until the colonies were visible. Colonies were then fixed in mix solution of 10% formaldehyde and 10% acetic acid at room temperature for 15 minutes, and stained with 0.1% crystal violet in methanol for 15 minutes. Then colonies were washed with PBS and dried at room temperature. Colony formation ability was quantitatively measured by dissolving crystal violet in 33.3% (v/v) acetic acid and detecting the absorbance (optical density, OD) at 600nm wavelength with SpectraMax plus384.

Tube formation assay

HUVEC were seeded in 6-well plates and pretreated with the indicated compounds for 24 hours. Then these cells were suspended in 10μL endothelial cell medium, seeded with a concentration of 1.5×10^4^ per well coated with 60μL matrigel in 96-well plates and maintained for 4-6 hours to form the tube. The formed tubes were captured by inverted fluorescence microscope (Olympus IX73, Tokyo, Japan).

Rat aortic ring sprouting assay

The aortas were isolated from rats and cut into about 1mm aortic rings. Then, each aortic ring was placed into 60μL precoated matrigel in 96-well plates overlayed with 100μL M199 medium supplemented with 20% FBS (Gibco, Thermo Fisher Scientific, Waltham, MA, USA) and 30μg/μL endothelial cell growth supplement, as well as treated with DMSO and corresponding compounds until the arterial rings started sprouting after 3~5 days. The microvessels from aortic ring were captured by inverted fluorescence microscope (Olympus IX73).

Transwell assay

HUVECs were maintained in medium alone or with various concentrations of indicated compounds for 24 hours, and then harvested and added to the upper chambers of 24-well transwell plates (8μm; Corning Costar Corp, NY, USA) with 100μL serum-free medium. 600μL ECM complete medium were added to the bottom chambers. After 12 hours, the migrated cells were fixed with 95% ethanol and dyed with 0.1% crystal violet. The images were captured by upright fluorescence microscope (Olympus B-51).

Tumor xenograft assay

All *in vivo* experiments were carried out according to the institutional ethical guidelines on animal care and were approved by the Institute of Animal Care and Use Committee at the Shanghai Institute of Materia Medica (No. 2016-04-DJ-21, 2020-04-DJ-56). HCC cell lines SMMC-7721, Huh7, JHH7 and Bel-7402，as well as patient-derived HCC cells were implanted subcutaneously in thymic BALB/c nude mice aged 4-6 weeks. The mice were randomly assigned to the control (12 mice) and treatment groups (six mice per treatment group). The treatment groups were received with AL3810, CYH33 and BYL719 daily by oral gavage. Tumors were measured twice per week by microcaliper and the individual relative tumor volumes (RTV) were calculated as follows: (½ × length × width^2^ of day n) / (½ × length × width^2^ of day 0). The therapeutic effect of the compounds was described with the volume ratio of treatment to control T/C (%) = 100%× (mean RTV of the treated group / mean RTV of the control group).

Immunohistochemistry assay

Paraffin embedded HCC xenograft tumor tissues were deparaffinized, and incubated with antibodies of CD31 (Abcam, Cambridge, Cambs, UK), α-SMA (CST, Beverly, MA, USA), and TUNEL in situ cell death detection Kit-POD (Roche, Basel, Switzerland) at 4°C overnight and secondary antibody (Envision detection system, DAKO) at room temperature for 30 minutes. Peroxidase activity was detected with diaminobenzidine.

Plasmids and transfection

mCherry and EGFP plasmids were purchased from Obio technology (Shanghai, China). The plasmid vehicles of mCherry and EGFP are pLenti-CMV-mCherry-3Flag-PGK-puro-WPRE and pLenti-EF1a-EGFP-P2A-Puro-CMV-MCS-3Flag. They were transfected into Bel-7402, SMMC-7721, MRC9 and WI38 cells by Lipofectamine 2000 (Invitrogen, Waltham, MA, USA) according to the manufacturer’s instructions.

Three-dimensional tumor spheroid formation

HCC cells SMMC-7721 and Bel-7402, as well as HUVEC, fibroblast MRC9 and WI38 cells were seeded alone or together in 100µL medium with the concentration of 8000, 8000, 2000, 2000 and 2000 cells per well respectively in black round bottom ultralow attachment spheroid 96-Microplate (#4520, Corning), and then cultured in 37°C and 5% CO_2_ for 3 days to allow the spheroid formation. The images were captured by inverted fluorescence microscope (Olympus IX73).

Three-dimensional tumor spheroid viability assay

Tumor spheroids were harvested and assayed according to the manufacturer’s recommendation of CellTiter-Glo® 3D cell viability assay kit (Promega, Madison, WI, USA). The same volume of reagent as the cell culture medium was added. After 5 minutes shaking, the cells were incubated at room temperature for 25 minutes. Then the luminescent signal was detected by synergy HT microplate reader (BioTek, Winooski, VT, US).

Three-dimensional tumor spheroid live/dead assay

Live/dead viability/cytotoxicity kit (Invitrogen, Waltham, MA, USA) was used to assess live/dead conditions of three-dimensional tumor spheroid after treatment with indicated compounds for 3 days. Briefly, three-dimensional tumor spheroids were rinsed twice with PBS cautiously, and incubated with corresponding assay reagents according to the manufacturer's protocol. The images were captured by inverted fluorescence microscope (Olympus IX73).

Statistical analyses

All data are presented as mean ± SD using GraphPad Prism software. Statistical significance was evaluated by student’s t-test for comparisons between two groups via GraphPad Prism software. P < 0.05 was considered statistically significant (*); P < 0.01 was considered very significant (**); and P < 0.001 was considered highly significant (***).


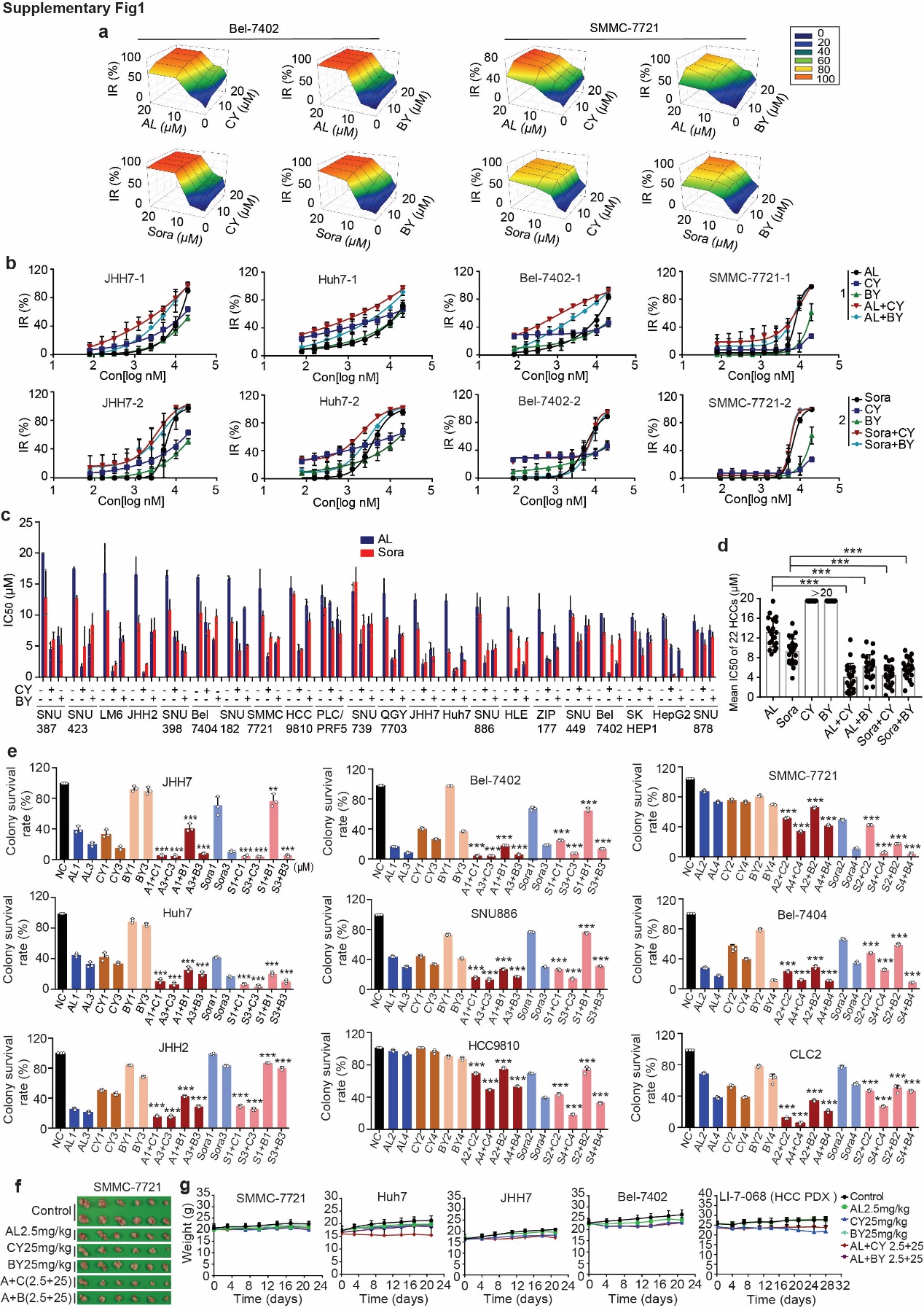


**Supplementary Figure. S1** **PI3Kα inhibitors sensitized AL3810 to inhibit multiple HCC tumors growth *in vitro* and *in vivo.* a** The “Heat” graphs were presented to display the combination efficacy of AL3810/sorafenib with CYH33 or BYL719 against Bel-7402 and SMMC-7721 cells. Bel-7402 and SMMC-7721 cells were exposed to AL3810 at serial concentrations in the presence or absence of CYH33 or BYL719 (5, 10, 15 and 20μM) for 3 days. X axis represents the concentration of AL3810 or sorafenib; Y axis represents the concentration of CYH33 or BYL719; Z axis represents the inhibition ratio under the co-treatment of AL3810 or sorafenib combined with CYH33 or BYL719. colors: corresponding inhibition ratio; IR: inhibition rate; AL: AL3810; CY: CYH33; BY: BYL719; Sora: sorafenib. **b** HCC cell lines JHH7, Huh7, Bel-7402 and SMMC-7721 were treated with AL3810, sorafenib, CYH33 and BYL719 at the starting concentration of 20μM with 2-fold dilution alone or combined for 3 days. The series of inhibition rate were determined by SRB assay and showed in dose-respond curves. Con: concentration. **c,** **d** IC_50_ values of AL3810/sorafenib combined with CYH33/BYL719 in 22 HCCs, under a series of concentration starting at 20μM in 2-fold dilution for 3 days. And the mean IC_50_ of 22 HCCs were presented in histogram with mean ± SD. **e** AL3810 or sorafenib combined with CYH33 or BYL719 synergistically suppressed colony formation, and the quantitative results were presented in histogram with mean±SD. **f** The representative tumor pictures of SMMC-7721 xenograft in each group were displayed. **g** The changes of mice body weight were presented in time-dependent curves presented with mean + SD and no distinct fluctuations were observed.


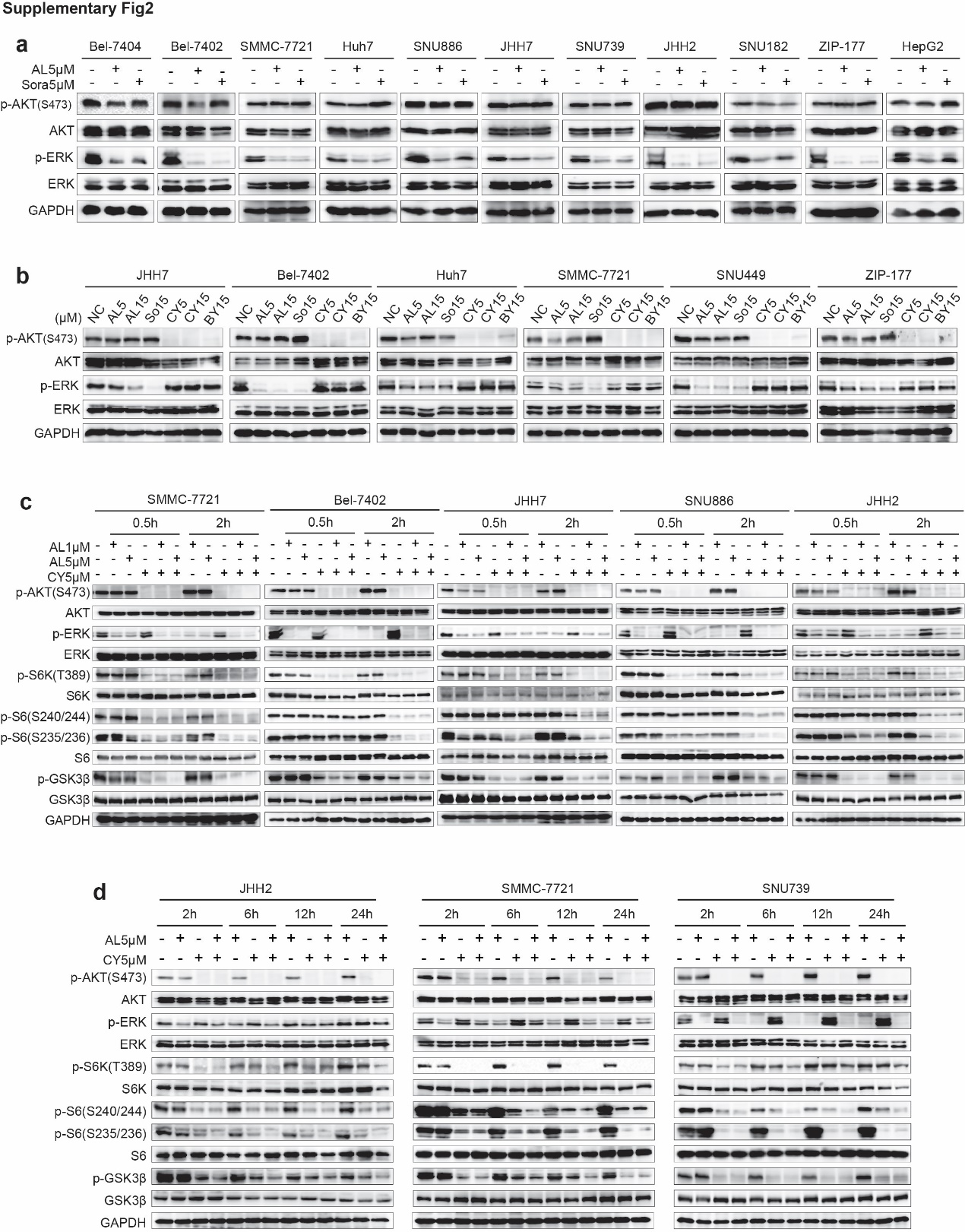


**Supplementary Figure. S2 Western blot analysis the expression levels of relative proteins in HCC cell lines treated with AL3810/Sorafenib PI3Kαis or both at indicated doses and time.** **a** 5μM sorafenib, same to 5μM AL3810, was applied to corroborate the coordinated inhibition for selectively targeting ERK phosphorylation rather than AKT phosphorylation in multiple HCCs. **b** Moreover, even when increasing higher concentration to 15μM, AL3810 and sorafenib still selectively inhibited ERK pathways, while CYH33 and BYL719 selectively suppressed AKT pathways. **c** Western blot analysis of the expression levels of downstream targeted genes in HCC cell lines SMMC-7721, Bel-7402, JHH7, SNU886 and JHH2, which treated with AL3810 in 1μM and 5μM alone or combined with 5μM CYH33. **d** Western blot analysis of the expression levels of downstream targeted genes in JHH2, SMMC-7721 and SNU739 treated with 5μM AL3810 and 5μM CYH33 in 2, 6, 12 and 24 hours.


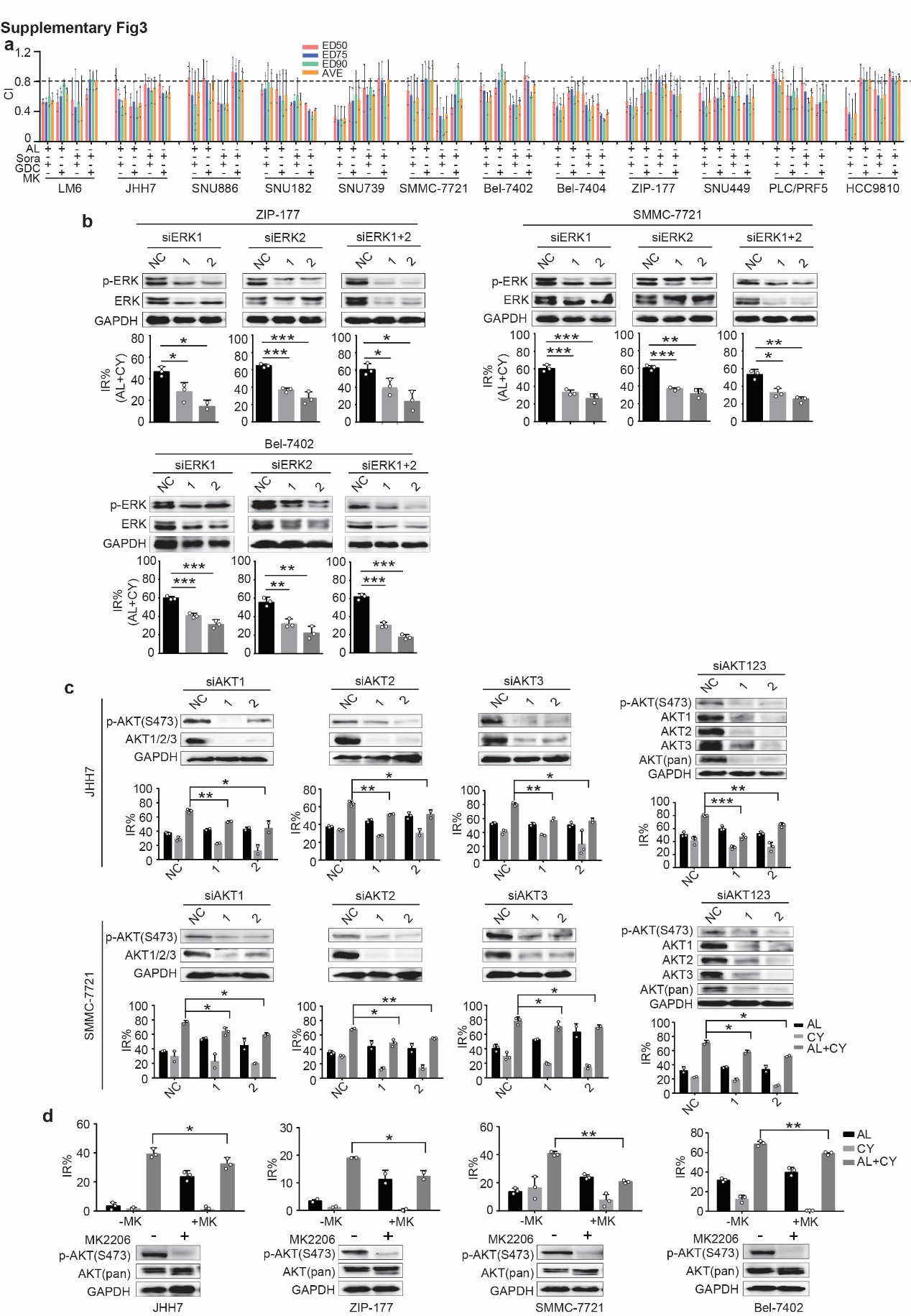


**Supplementary Figure. S3 AL3810 and PI3Kα inhibitors blocked MAPK-ERK and PI3K-AKT pathways in HCCs. a** The CI values of multiple HCC cell lines were calculated. Cells were treated with AL3810/sorafenib and AKT inhibitors GDC0068 /MK2206 alone or in combined with a series of concentration starting at 20μM in 2-fold dilution for 3 days. GDC: GDC0068; MK: MK2206. **b, c** Western blot confirming the silencing efficiency of siERK1, siERK2, siERK (1+2), or siAKT1, siAKT2, siAKT3, siAKT (1+2+3) (up panel), meanwhile the combined inhibition rate (IR) for AL3810 and CYH33 in HCC cells were detected by SRB assays (down panel). **d** The combination inhibition rate (IR) in MK2206 pre-treated HCC cells (up panel), as well as the phosphorylation of AKT identified by western blot (down panel).


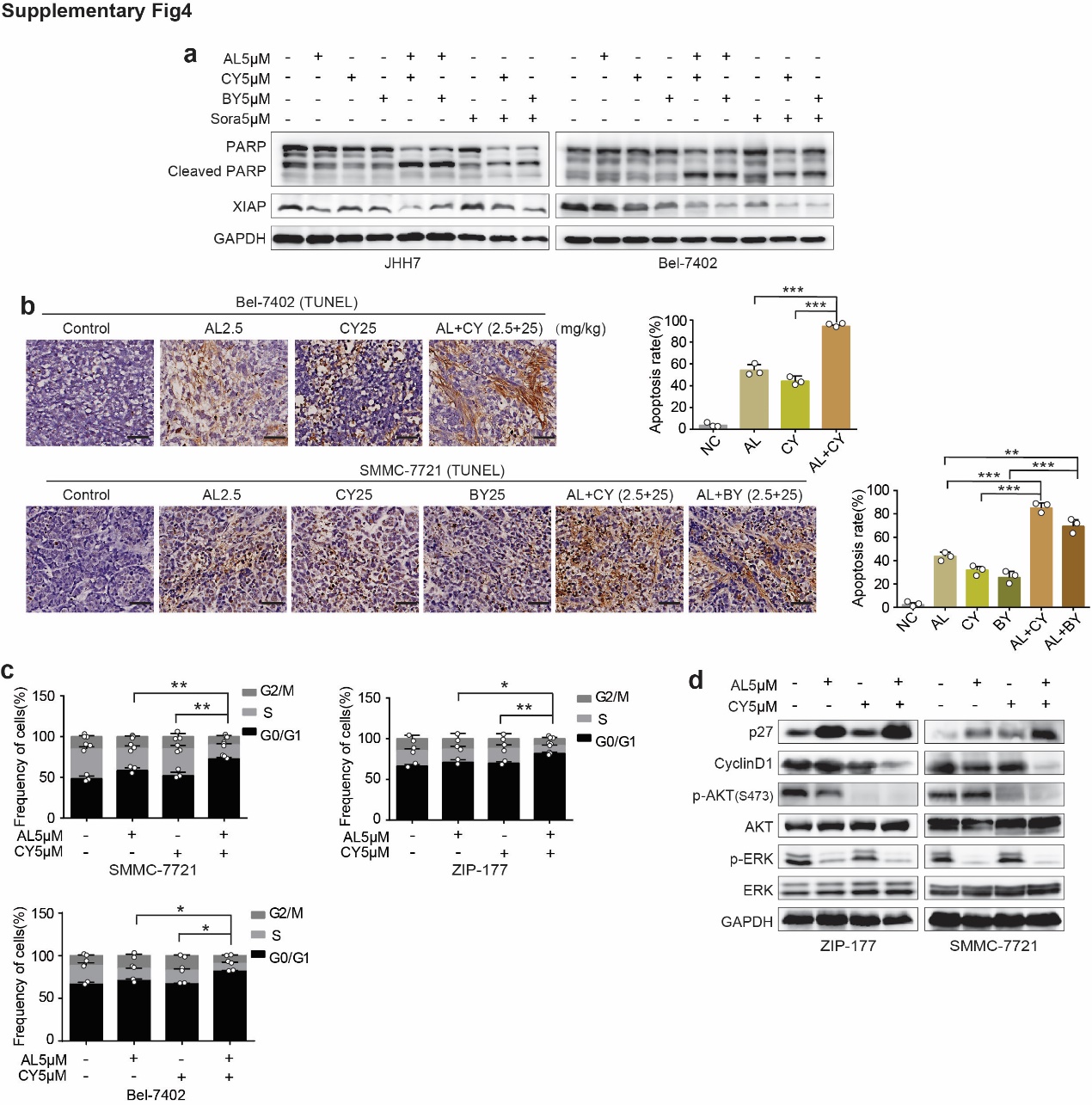


**Supplementary Figure. S4 AL3810 and PI3Kα inhibitors synergistically induce apoptosis and retard G1 phase in HCC.** **a** The expression of apoptosis related proteins PARP, cleaved PARP and XIAP in JHH7 and Bel-7402 cells were examined by western blot following treatment with AL3810/ sorafenib combined with CYH33/BYL719 for 72 hours. **b** Representative immunohistochemical stanning images of TUNEL derived from Bel-7402 and SMMC-7721 xenografts (left panel) and the quantified results were displayed in histogram with mean ± SD (right panel); Scale bar indicates 50μm. **c** The quantitative results of cell cycle for ZIP-177, SMMC-7721 and Bel-7402 cells were presented in histogram with mean ± SD. **d** The expression level of cell cycle related proteins p27 and CyclinD1 in ZIP-177 and SMMC-7721 cells were validated by western blot.


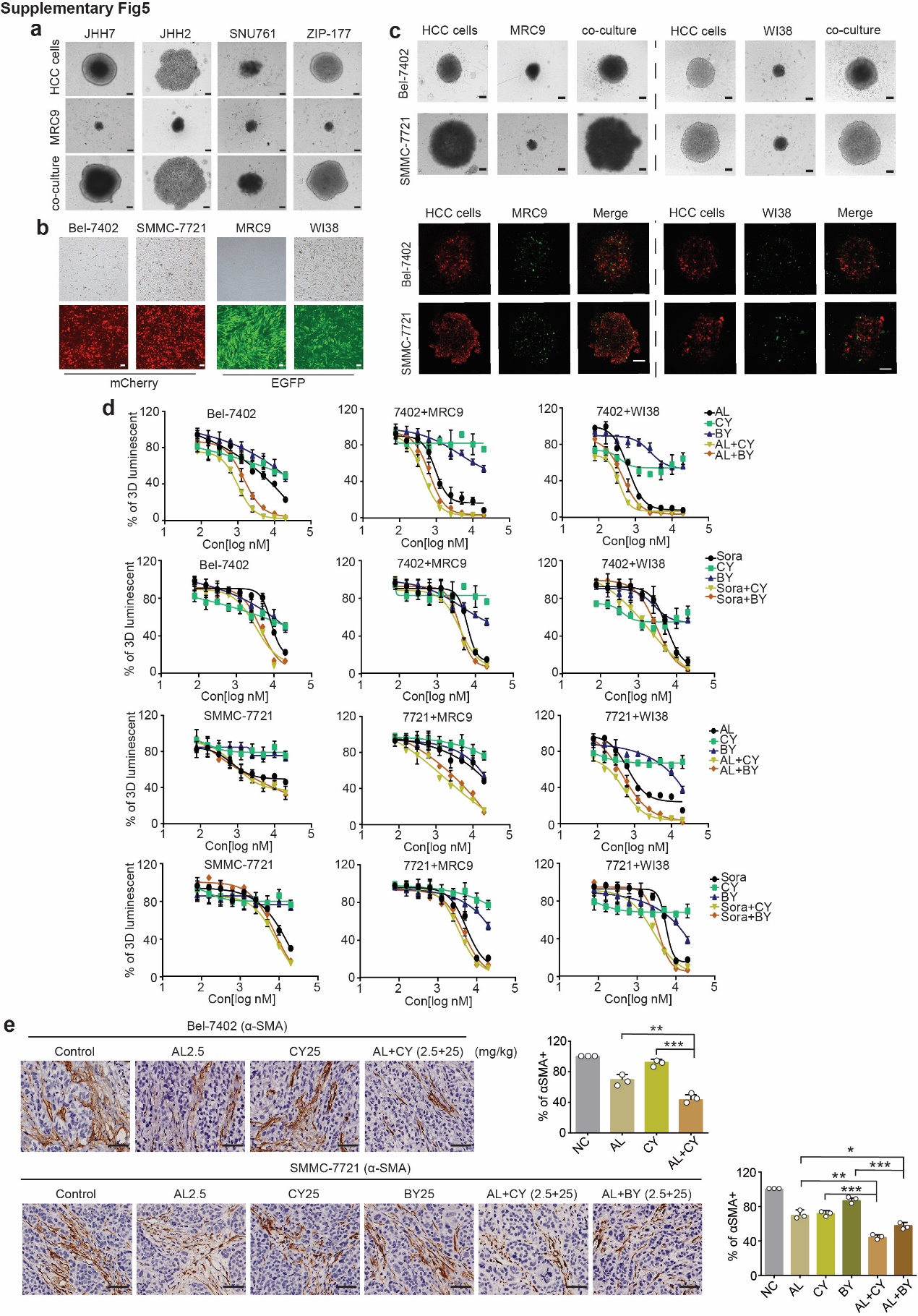


**Supplementary Figure. S5 Combination therapy of AL3810 and PI3Kα inhibitors inhibited three-dimensional tumor spheroid survival of HCCs co-cultured with fibroblast.** **a** The establishment and validation of three-dimensional tumor spheroid for more HCC cell lines including JHH7, JHH2, SNU761 and ZIP-177. Scale bar indicates 100μm. **b** Representative fluorescence pictures of Bel-7402 and SMMC-7721 cells as well as MRC9 and WI38 cells transfected with mcherry and EGFP fluorescence respectively. Scale bar indicates 100μm. **c** The establishment and validation of 3D tumor spheroid for HCC cells Bel-7402 and SMMC-7721 with fibroblast WI38 and MRC9 cells co-cultured in a proportion of 4:1 (up panel) and the location for three-dimensional co-culture after transfected with mcherry and EGFP fluorescence (down panel). Scale bar indicates 100μm. **d** The 3D tumor spheroids with Bel-7402 and SMMC-7721 cells alone or with mixtures (Bel-7402+MRC9/WI38 cells or SMMC-7721+MRC9/WI38 cells) were treated with AL3810/sorafenib combined with CYH33/BYL719 for 3 days. And the viability of the 3D tumor spheroids was detected using CellTiter-Glo 3D cell viability assay kit. **e** Representative immunohistochemical staining images of α-SMA derived from SMMC-7721 and Bel-7402 xenograft tissues (left panel) and the quantified results were displayed in histogram with mean ± SD (right panel); Scale bar indicates 50μm.


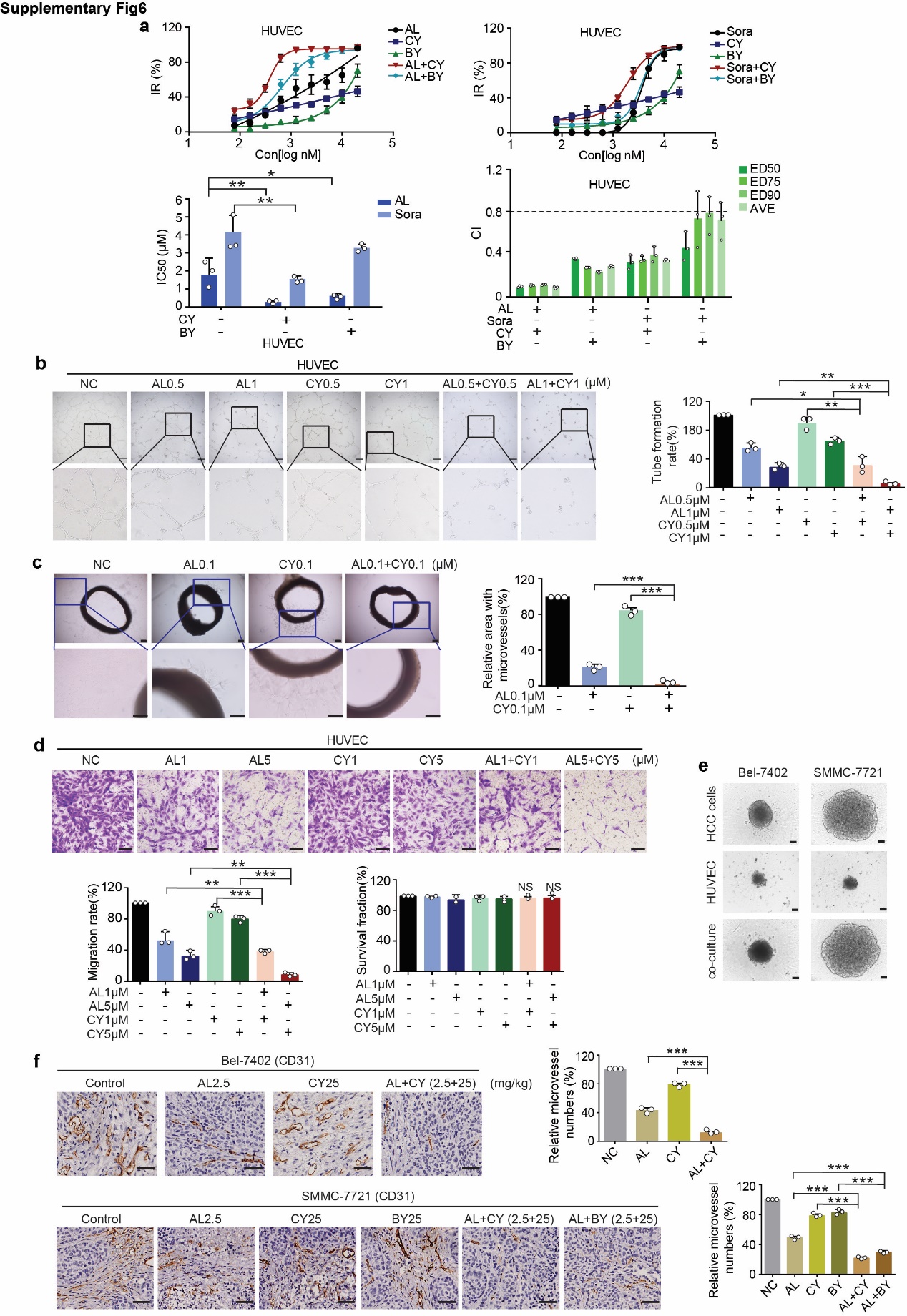


**Supplementary Figure. S6 AL3810 synergized with PI3Kα inhibitors to suppress angiogenesis. a** HUVEC were incubated with AL3810/sorafenib combined with CYH33/BYL719 at the starting concentration of 20μM with 2-fold dilution for 3 days, and the synergistic effects were characterized by CI values. **b** The ability of tube formation was potently impaired by treatment of AL3810 combined with CYH33 (left panel) and the quantified results were showed in histogram with mean ± SD (right panel). Scale bar indicates 200μm. **c** Rat aortic ring sprouting assay reflected the synergistic effects on angiogenesis with treatment of AL3810 combined with CYH33 (left panel) and the quantified results were presented in histogram with mean ± SD (right panel). Scale bar indicates 200μm. **d** HUVECs were treated with AL3810 and CYH33 alone or combined in transwell chamber for 12 hours (up panel). And the migrated cells, as well as cell survival levels were detected and presented in quantitative histogram with mean ± SD (down panel). Scale bar indicates 100μm. **e** Bel-7402 and SMMC-7721 cells were co-cultured with HUVECs to form the three-dimensional tumor spheroid, Scale bar indicates 100μm. **f** Representative immunohistochemical staining images of CD31 derived from SMMC-7721 and Bel-7402 xenograft tissues (left panel) and the quantified results were presented in histogram with mean ± SD (right panel); Scale bar indicates 50μm.

**Supplementary table S1. Hepatocellular carcinoma cell lines and culture conditions**

| **Cell lines** | **Culture condition** | **Source** |
| --- | --- | --- |
| SNU423 | 1640+10%FBS | ATCC (Manassas, VA, USA) |
| JHH2 | 1640+10%FBS | ATCC |
| JHH7 | 1640+10%FBS | ATCC |
| SNU182 | 1640+10%FBS | ATCC |
| SNU739 | 1640+10%FBS | ATCC |
| ZIP-177 | 1640+10%FBS | ATCC |
| SNU387 | 1640+10%FBS | ATCC |
| SNU886 | 1640+10%FBS | ATCC |
| SNU398 | 1640+10%FBS | ATCC |
| SNU449 | 1640+10%FBS | ATCC |
| SNU878 | 1640+10%FBS | ATCC |
| HCC9810 | 1640+10%FBS | ATCC |
| SK-HEP-1 | EMEM+10%FBS | ATCC |
| HepG2 | EMEM+10%FBS | ATCC |
| WI38 | EMEM+10%FBS | ATCC |
| MRC9 | EMEM+10%FBS | ATCC |
| HLE | DMEM+10%FBS | ATCC |
| Huh7 | DMEM+10%FBS | Institute of Biochemistry and Cell Biology (Shanghai, China) |
| Bel-7402 | 1640+10%FBS | Institute of Biochemistry and Cell Biology |
| SMMC-7721 | 1640+10%FBS | Institute of Biochemistry and Cell Biology |
| Bel-7404 | 1640+10%FBS | Institute of Biochemistry and Cell Biology |
| QGY-7703 | 1640+ sodium pyruvate+ glucose +10%FBS | Institute of Biochemistry and Cell Biology |
| CLC2 | 1640+10%FBS+1%ITS+40%ng/mL EGF | Institute of Biochemistry and Cell Biology |
| HUVEC | Endothelial cell medium | Allcells (Silicon Valley, CA, USA) |
| LM6 | DMEM+10%FBS | Zhongshan Hospital (Shanghai, China) |
| PLC/PRF5 | DMEM+10%FBS | CrownBio (Silicon Valley, CA, USA） |

**Supplementary table S2. antibodies**

| **Antibodies** | **Source** | **Identifier** |
| --- | --- | --- |
| P-AKT (S473) | CST (Beverly, MA, USA) | Cat#:4060S |
| AKT (pan) | CST | Cat#:4691S |
| AKT1 | CST | Cat#:2967S |
| AKT2 | CST | Cat#:3063S |
| AKT3 | CST | Cat#:14982S |
| P-ERK | CST | Cat#:4370S |
| ERK | CST | Cat#:4695S |
| P-S6K (T389) | CST | Cat#:97596S |
| S6K | CST | Cat#:2708S |
| P-S6 (S240/244) | CST | Cat#:5364S |
| P-S6 (S235/236) | CST | Cat#:4858S |
| S6 | CST | Cat#:2217S |
| P-GSK3β (S9) | CST | Cat#:5558S |
| GSK3β | CST | Cat#:12456S |
| GAPDH | CST | Cat#:5174S |
| PARP | CST | Cat#:9532S |
| XIAP | CST | Cat#: 14334S |
| P27 | CST | Cat#:3686S |
| CyclinD1 | CST | Cat#:2978S |
| α-SMA | CST | Cat#:56856 |
| CD31 | Abcam (Cambridge, Cambs, UK) | Cat#: ab182981 |

**Supplementary table S3. reagents**

| **Reagents** | **Source** | **Identifier** |
| --- | --- | --- |
| Fetal bovine serum (FBS, Gibco) | Thermo Fisher Scientific  (Waltham, MA, USA) | Cat#:10099141 |
| Non-essential amino acids (NEAA) | Thermo Fisher Scientific | Cat#:11140050 |
| Sodium pyruvate | Thermo Fisher Scientific | Cat#:11360070 |
| 4',6-diamidino-2-phenylindol (DAPI) | Thermo Fisher Scientific | Cat#: D1306 |
| Hematoxylin[and eosin](https://www.beyotime.com/he-staining-kit.htm) (HE) | Thermo Fisher Scientific | Cat#: 7211 |
| Lipofectamine RNAiMAX | Thermo Fisher Scientific | Cat#: 13778150 |
| TRIzol reagent | Thermo Fisher Scientific | Cat#: 10296028 |
| Diaminobenzidine (DAB) | Sigma  (Darmstadt, Hesse, Germany) | Cat#: D8001 |
| Dimethylsulfoxide (DMSO) | Sigma | Cat#: BML-KI597-0400 |
| Dithiothreitol (DTT) | Sigma | Cat#: 43815 |
| Sulforhodamine B (SRB) | Sigma | Cat#: 341738 |
| Bovine serum albumin (BSA) | Sigma | Cat#: B2064 |
| Normal goat serum | JACKSON  (Lancaster, PA, USA) | Cat#: 005-000-121 |
| Peroxidase affinipure goat anti-rabbit IgG | JACKSON | Cat#: 111-035-0030 |
| Protease inhibitor | Roche (Basel, Switzerland) | Cat#: 04693159001 |
| Phosphatase inhibitor | Roche | Cat#:04906837001 |
| TUNEL in situ cell death detection kit-POD | Roche | Cat#: 11684817910 |
| RNAse A | Beyotime Biotechnology  (Shanghai, China) | Cat#: ST576 |
| Propidium iodide (PI) | Beyotime Biotechnology | Cat#: ST511 |
| Phosphate buffer saline (PBS) | Beyotime Biotechnology | Cat#: C0221A |
| RIPA lysis buffer | Beyotime Biotechnology | Cat#: P0013B |
| Paraformaldehyde (PFA） | Beyotime Biotechnology | Cat#: P0099 |
| Human EGF | PeproTech | Cat#: AF-100-15 |
| Endothelial cell growth supplement (ECGS) | MT-BIO (Shanghai, China) | Cat#:1166 |
| Matrigel | Corning (New York, NY, USA) | Cat#:356237 |
| Sodium dodecyl sulfate (SDS) | Biosharp (Beijing, China) | Cat#: BS028B |
| Insulin-transferrin-selenium (ITS) | Life (Carlsbad, CA, USA) | Cat#: 51300-044 |
| CellTiter-Glo 3D cell viability assay kit | Promega (Madison, WI, USA) | Cat#: G9682 |
| Live/dead viability/cytotoxicity kit | Invitrogen (Waltham, MA, USA) | Cat#: L3224 |
| Lipofectamine 2000 | Invitrogen | Cat#: #11668019 |
| Human phospho-MAPK array kit | R＆D Systems  (Minneapolis, MN, USA) | Cat#: ARY002B |
| Annexin Ⅴ-FITC/PI double staining apoptosis kit | Vazyme Biotech  (Nanjing, China) | Cat#: A211-01 |

**Supplementary table S4. siRNA sequences in RNA interference**

| **Gene** | **siRNA sense (5’-3’)** | **siRNA antisense (5’-3’)** |
| --- | --- | --- |
| AKT1-1 | GCACCUUCAUUGGCUACAATT | UUGUAGCCAAUGAAGGUGCTT |
| AKT1-2 | GAGGCCAAGUCCUUGCUUUTT | AAAGCAAGGACUUGGCCUCTT |
| AKT2-1 | GCUCCUUCAUUGGGUACAATT | UUGUACCCAAUGAAGGAGCTT |
| AKT2-2 | GGUUCUUCCUCAGCAUCAATT | UUGAUGCUGAGGAAGAACCTT |
| AKT3-1 | GGAUGCCUCUACAACCCAUTT | AUGGGUUGUAGAGGCAUCCTT |
| AKT3-2 | GCUUUCAGGGCUCUUGAUATT | UAUCAAGAGCCCUGAAAGCTT |
| ERK1-1 | CCUUCGAACAUCAGACCUATT | UAGGUCUGAUGUUCGAAGGTT |
| ERK1-2 | GAGAUGUCUACAUUGUGCATT | UGCACAAUGUAGACAUCUCTT |
| ERK2-1 | GUGAUCUCAAGAUCUGUGATT | UCACAGAUCUUGAGAUCACTT |
| ERK2-2 | GUCCAUUGAUAUUUGGUCUTT | AGACCAAAUAUCAAUGGACTT |
